# Supplementary material for: Ear pinnae in a neotropical katydid (Orthoptera: Tettigoniidae) function as ultrasound guides for bat detection
Source: eLife. 2022 Sep 28;11:e77628. doi: 10.7554/eLife.77628 (PMC9519150; doi:10.7554/eLife.77628)
Supplement: Transparent reporting form [file elife-77628-transrepform1.docx]

***eLife’s* transparent reporting form**

We encourage authors to provide detailed information *within their submission* to facilitate the interpretation and replication of experiments. Authors can upload supporting documentation to indicate the use of appropriate reporting guidelines for health-related research (see [EQUATOR Network](http://www.equator-network.org/%20)), life science research (see the [BioSharing Information Resource](https://biosharing.org/)), or the [ARRIVE guidelines](http://www.plosbiology.org/article/info:doi/10.1371/journal.pbio.1000412) for reporting work involving animal research. Where applicable, authors should refer to any relevant reporting standards documents in this form.

If you have any questions, please consult our Journal Policies and/or contact us: [editorial@elifesciences.org](mailto:editorial@elifesciences.org).

**Sample-size estimation**

- You should state whether an appropriate sample size was computed when the study was being designed
- You should state the statistical method of sample size computation and any required assumptions
- If no explicit power analysis was used, you should describe how you decided what sample (replicate) size (number) to use

Please outline where this information can be found within the submission (e.g., sections or figure legends), or explain why this information doesn’t apply to your submission:

Doesn’t apply: It was also not possible to calculate samples prior to the commencement of the data collection, as we had no reliable estimation of effect sizes and the collection of multiple repeated measures data points per individual specimen makes sample size calculations hard. We designed the experiment with numerous measurements per specimen to take into account the limited availability of neotropical katydids during the pandemic and consequent travel restrictions. Experiment design incorporated appropriate statistical models.

**Replicates**

- You should report how often each experiment was performed
- You should include a definition of biological versus technical replication
- The data obtained should be provided and sufficient information should be provided to indicate the number of independent biological and/or technical replicates
- If you encountered any outliers, you should describe how these were handled
- Criteria for exclusion/inclusion of data should be clearly stated
- High-throughput sequence data should be uploaded before submission, with a private link for reviewers provided (these are available from both GEO and ArrayExpress)

Please outline where this information can be found within the submission (e.g., sections or figure legends), or explain why this information doesn’t apply to your submission:

This information is found in the *Materials and Methods* subsections for individual sets of experiments. Specific subsections:

*Specimens* (line 589), *Morphological studies of the ear* (line 678), *Bat and insect call recordings* (line 696) *Acoustics measurements of synthetic 3D-printed scaled ear models* (line 711), *Behavioural and tympanal response to broadband stimulation* (line 786), *neural audiograms* (line 804), and explained further when referenced in the *Supplementary Materials and Methods Text*.

Information is also provided in legends: Figure (Figs. 2 and 5) and Tables S1-S3.

**Statistical reporting**

- Statistical analysis methods should be described and justified
- Raw data should be presented in figures whenever informative to do so (typically when N per group is less than 10)
- For each experiment, you should identify the statistical tests used, exact values of N, definitions of center, methods of multiple test correction, and dispersion and precision measures (e.g., mean, median, SD, SEM, confidence intervals; and, for the major substantive results, a measure of effect size (e.g., Pearson's r, Cohen's d)
- Report exact p-values wherever possible alongside the summary statistics and 95% confidence intervals. These should be reported for all key questions and not only when the p-value is less than 0.05.

Please outline where this information can be found within the submission (e.g., sections or figure legends), or explain why this information doesn’t apply to your submission:

Statistical reporting is found in the *Materials and Methods* subsection *Statistical analyses* and Table S1.

(For large datasets, or papers with a very large number of statistical tests, you may upload a single table file with tests, Ns, etc., with reference to sections in the manuscript.)

**Group allocation**

- Indicate how samples were allocated into experimental groups (in the case of clinical studies, please specify allocation to treatment method); if randomization was used, please also state if restricted randomization was applied
- Indicate if masking was used during group allocation, data collection and/or data analysis

Please outline where this information can be found within the submission (e.g., sections or figure legends), or explain why this information doesn’t apply to your submission:

Groups are samples of live specimens and 3D generated ears (in print or simulations) allocated to experimental group.

**Additional data files (“source data”)**

- We encourage you to upload relevant additional data files, such as numerical data that are represented as a graph in a figure, or as a summary table
- Where provided, these should be in the most useful format, and they can be uploaded as “Source data” files linked to a main figure or table
- Include model definition files including the full list of parameters used
- Include code used for data analysis (e.g., R, MatLab)
- Avoid stating that data files are “available upon request”

Please indicate the figures or tables for which source data files have been provided:

Statistical models (in particular LMMs) are described in the *Materials and Methods* subsection *Statistical analyses* and intended to be coded from description. Experimental data (LDV recordings), numerical simulations, Comsol model files, and µ-CT stereolithography files (in .stl format) are available in Dryad (<https://doi.org/10.5061/dryad.k0p2ngf8x>; deposited 9 September 2021).
